# Finding the key to the black box of board diversity and firm performance: A mediating effect analysis of technological innovation

**DOI:** 10.3389/fpsyg.2022.914215

**Published:** 2022-07-26

**Authors:** He Di, Jiaji An, Meifang Yao

**Affiliations:** School of Business and Management, Jilin University, Changchun, China

**Keywords:** board diversity, firm performance, technological innovation, mediating effect, resource dependence theory

## Abstract

A growing body of research has focused on the relationship between board diversity and firm performance. A series of empirical literatures have also examined a significant positive correlation between the two. But these results only demonstrate the relationship between the input of ‘diversity’ and the output of ‘firm performance’. Such research is more of a black box because board diversity must act on certain strategies or decisions to affect firm performance. Some scholars try theoretical analysis with the purpose of opening the black box. In order to verify the relevant theoretical analysis results, this study uses the mediating effect analysis model in the field of psychology, through multiple regression, impulse analysis, variance decomposition and other methods, to thus empirically test the mediating effect of technological innovation in the process of board diversification promoting corporate performance. We found that board diversity can improve firm performance by enhancing the level of technological innovation. Among them, technological innovation has played a complete mediating role in the diversity of board members’ functional and occupational background, and played a partial mediating role in the diversification of directors’ part-time jobs. Technological innovation is a key indicator bridging board diversity and firm performance. This study can explore and explain the inner workings of the significant relationship between board diversity and firm performance, and link research findings on similar phenomena. The research results may make the existing board governance theories more systematic, expand the extension of theoretical research, and provide some empirical research references for academics and practitioners.

## Introduction

With the deepening of the research on the modern corporate governance mechanism, the research on “people” is also gradually focused. From the research on the dichotomy of owners and managers to parallel analysis, to the optimization of corporate governance mechanisms, then to the refocusing of board governance, the research path continues to develop in depth. At present, the research on corporate governance has focused on the personal characteristics of decision-making level and management level. Board capital is one area of these. It reveals the significant impact that individuals have on the organization as a whole (Hillman et al., 2000). Hillman and Dalziel (2003) introduced the concept of capital into board governance behavior based on corporate governance theory, namely “board capital.” They believed that due to differences in board capital, two companies with similar board structures have significant differences in their strategic decisions and effects. Since then, researchers have continued to focus on the new perspective of directors’ personal characteristics to study the behavior and effectiveness of board governance. Some scholars also empirically tested the positive effect of board diversity on corporate governance and firm performance (Guest, 2019; Knyazeva et al., 2021). These instructive research results have attracted the attention of some companies and regulators. For example, given the importance of board diversity, the Singapore Stock Exchange requires issuers to formulate board diversity policies from 1st January 2022 to address diversity-related aspects such as gender, skills and experience. Issuers are also required to disclose details of their board diversity policy and diversity goals, plans, timelines and progress in their annual reports. In conclusion, since research on board capital is so lucrative, both in theory and in practice, this arouses our research interest. However, board diversity is a potential internal cause of corporate performance improvement, and the causal relationship between the two needs to be bridged by some kind of strategy or decision variable. Most of the previous literature only proves the input–output relationship between the two, which is more like a “black box” study (Hillman, 2015). Different from the existing literature, this study argues that a novel variable should be added to the traditional research relationship. In our view, technological innovation is the X factor and a key to the black box. On one hand, the diversification of board capital will improve the level of innovation strategy and the effect of strategy implementation. On the other hand, it has basically reached a consensus that technological innovation can improve company performance. Therefore, there is a theoretical logic of the mediating effect between board diversity, technological innovation and corporate performance. In order to open the black box, improve the economic meaning of the research results, and explore the internal mechanism and causal relationship, this study starts from the perspective of technological innovation, and deeply analyzes its mediating effect in the process of board diversity affecting firm performance. This brand-new test of the relationship between mediation effects may also become the special value and main contribution of this paper. Our main research questions include:

- Does board diversity affect the level of technological innovation and performance of companies?
- What is the internal mechanism of this “black box”? Does technological innovation play a significant mediating effect?
- Could technological innovation become the link between board diversity and firm performance and the key to unlocking the black box?

## Literature review

Board governance is an important part of corporate governance and is in a special position. It not only fulfills the fiduciary responsibility entrusted by the shareholders’ meeting, but also fulfills the responsibility of entrusting for the management. Therefore, research on board functions and the resources they provide has become a central issue in board governance research (Dato et al., 2020). The basic function of the board of directors is to select, monitor, reward or punish managers. However, with the deepening of research, more theories such as resource dependence theory have been introduced into the field of board governance research. Some scholars have pointed out that oversight is the main function of the board of directors, but it is not the only function. Boards can also help companies improve strategic and operational performance by providing advice, securing external resources, developing management capabilities, and conducting crisis management (Shaukat and Trojanowski, 2018; Seijts et al., 2019). The existing research perspectives on board governance can be divided into macro and micro levels. The research at the macro level focuses on the role of the board of directors, the scale, and the proportion of outside directors to describe the running state of the entire board of directors. At the micro level, more consideration is given to the characteristics of board members, that is, the impact of board capital on firm performance (Conyon, 2014). Based on the theme of this paper, we will focus on the research from the micro level.

### Concept evolution from board capital to board diversity

Before the concept of board capital was raised, some scholars have studied the variables belonging to the category of board capital such as age and gender from various perspectives (Zahra and Pearce, 1989; Dupree and Quarterman, 2001). But since Hillman and Dalziel (2003) first introduced the concept of board capital into the corporate governance system, theoretical research on board capital has attracted more and more attention. Scholars have mainly studied board capital from two dimensions: human capital and social capital (Fischer et al., 2009; Johnson et al., 2013; Sun et al., 2016). Hillman and Dalziel (2003) believed that board capital is the sum of human capital such as professional knowledge, skills and experience that directors can provide, and social capital such as internal and external networks. Board capital comes from the personal characteristics of board members. By exercising resource provision function, and supervision and control function through the governance of board of directors can effectively reduce the company’s dependence on external resources, thereby bringing endogenous and heterogeneous competitive advantages. Chen (2014) argued that board capital can measure the ability of board members to provide resources to the company. Among them, human capital of the board of directors refers to the general term of the professional knowledge, skills and experience brought by the members to the organization; social capital of the board of directors refers to the internal and external relationship networks owned by directors and the potential resources brought by these relationship networks. Further research suggests that board human capital and social capital are inseparable, and there is an interaction of roles (Davidsson and Honig, 2003). With the further in-depth study of board capital, the alternative indicators of its variables are also evolving. Haynes and Hillman (2010) analyzed the impact of board capital on corporate strategic decisions in different periods and at different levels through case studies, and divided board capital into two dimensions: capital breadth and capital depth. Among them, the breadth of board capital refers to the degree of heterogeneity that board members can provide enterprises with various resources, which can also be understood as the degree of diversification. It specifically includes: (1) the heterogeneity level of directors’ occupational background; (2) the heterogeneity level of directors’ functional background; and (3) the heterogeneity level of directors’ part-time job situation. This classification has been adopted by many scholars (Muttakin et al., 2018; Jing et al., 2021; Barroso-Castro et al., 2022). Since then, board diversity has officially become an important indicator to measure the governance structure of the board of directors.

### Board diversity

Based on the theory of organizational heterogeneity, Baum et al. (2000) believed that the greater the diversity of organizational members, the more dynamic and advantageous for the entire organization. Wang and Xia (2021) investigated the heterogeneity of CEO management teams. They confirmed that as the CEO and his management team were more differentiated, the company became more competitive in the market and more adaptable to the industry environment. The research from Mollah et al. (2021) on the supervision ability of independent directors showed that the greater the difference of independent directors, the more effective their supervision were. Emuron and Tian (2020) found that when the board of directors had members who serve in financial institutions, it was often more unlikely for companies to get into financial distress. Because they cannot only benefit the company through professional knowledge, skills, etc., but also gain potential financing advantages through their personal network. Since a highly heterogeneous leadership team can help companies improve strategic decision-making in a dynamically changing environment, complex corporate operational behavior need to be planned by a leadership team with cognitive heterogeneity (Somech, 2006), thereby enhancing organizational creativity and competitive advantage in adapting and improving firm performance (Coles et al., 2008). Therefore, board diversity can improve the ability of the board of directors to perform the function of providing resources and build the internal differentiation advantage of the enterprise, and then by acting as the highest decision maker of the enterprise, promote the improvement of the company’s performance (Islam et al., 2022). Bossaller et al. (2017) argued that board members can provide companies with resources other than capital, such as expertise, experience, and strategic advice. Through these differentiated resources of the functional backgrounds of board members, companies can gain useful advice on organizational innovation. Due to the addition of different knowledge and viewpoints, the knowledge and expertise of the decision-making level tended to be heterogeneous, which can better identify innovation opportunities, broaden innovation horizons and contribute to the generation and implementation of high-quality innovation decisions. On the contrary, if the professional background of the board members became homogeneous, the board of directors tended to implement conservative strategies and was not good at identifying and discovering new opportunities, which was not conducive to the company’s technological innovation (Wang and Xia, 2021).

### Technological innovation

The impact of technological innovation on firm performance has been continuously developed since Schumpeter’s innovation theory was raised. Adner and Kapoor (2010) pointed out that technological innovation activities of enterprises are an important factor affecting their business performance, and verified the positive effect of technological innovation on enterprise performance through empirical tests. Ahuja and Katila (2001) used future development opportunities as a dependent variable to explore the relationship between technological innovation and it. They pointed out that technological innovation is significantly positively related to the future development opportunities of enterprises. The research results of Camison and Villar-Lopez (2014) showed that the intensity of technological innovation had a positive impact on firm performance, and this impact had a certain time lag. Ko and Kim (2021) randomly selected more than 3,000 companies to conduct a questionnaire survey and found that the level of technological innovation of enterprises is significantly positively correlated with their performance. Xie et al. (2018) studied the innovation output performance of high-tech enterprises and believed that R&D investment had a positive impact on both the short-term and long-term financial performance of the company. Xu et al. (2019) randomly screened and identified 241 manufacturing companies in China, and used 8 indicators such as return on sales to measure corporate performance through questionnaires, and studied the level of technological innovation of companies. Their conclusion was that technological innovation significantly improved firm performance. In general, the positive effect of technological innovation on firm performance has been proved by a large number of theoretical analysis and empirical research literature. Based on the above, we believed that the influence of the diversification of the board structure on the company’s performance is not direct, and it should be the potential internal cause of the company’s performance improvement. This internal factor is reflected in the effectiveness of board diversity, which directly affects a certain decision of the company, thereby improving the company’s performance. Simply studying the effect of board capital on corporate performance will inevitably overlook the intermediate links and cannot open the “black box” of the mechanism of action (Klarner et al., 2020). Since the existing literature has little research on the internal mechanism of board diversity affecting corporate performance, this paper takes technological innovation as the key to unlocking the black box and focuses on the relationship between board diversity, technological innovation and corporate performance. We connected two stages of research, that is, resource provision (board diversity)—decision execution (technical innovation), decision execution (technical innovation)—effect output (firm performance), and then fully demonstrated the internal mechanism between board diversity and firm performance.

## Theoretical framework

### Theoretical basis of board governance

**Figure 1:**
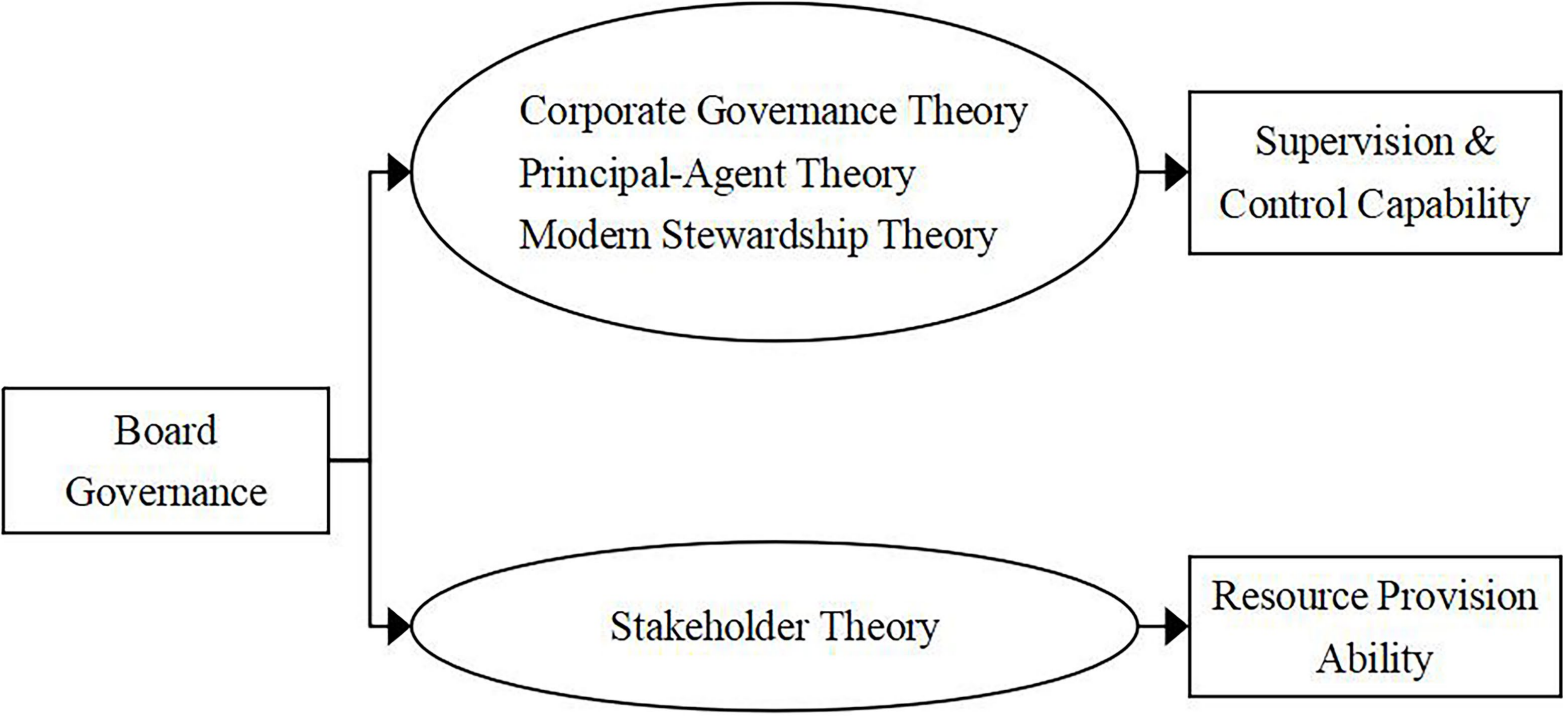
Theoretical framework of board governance.

Since the 1980s, the study of board governance has become a hot spot in the field of corporate governance research. With the continuous establishment and improvement of relevant theoretical systems and the continuous maturity of corporate governance-related research, results of research on the structure and characteristics of the board of directors were gradually produced. Zahra and Pearce (1989) established an overall theoretical framework for board governance for the first time. Later scholars continued to revise and improve it on this basis, and gradually formed the theoretical basis of the current board governance. After reviewing a large number of relevant literatures, Di (2018) pointed out that the basic theories of board governance mainly include corporate governance theory, principal-agent theory, modern stewardship theory and stakeholder theory. Among them, the first three theories mainly focused on the board’s ability to supervise and control the company, and discussed how the board of directors plays a role in the company. The stakeholder theory extended board governance to the outside of the company, focusing on the analysis of the resources provided by directors and stakeholders to the company. This theory held that companies built ties with the government by paying taxes in accordance with the law and seeking policy support, and at the same time, they had social responsibilities for the development of their communities and local regions. Therefore, shareholders cannot be regarded as the sole owner of the company, and the interests of internal and external stakeholders should also be considered (Kaler, 2006). At the same time, in the process of maximizing firm performance, the joint participation of internal and external stakeholders was required (Freeman et al., 2020). Companies should enhance the broad participation of the board of directors, attract non-shareholders such as employees, creditors and community representatives, and ensure their voice and voting rights (Polat, 2021). The theoretical support of the stakeholder theory for this paper was to promote more stakeholders into the board of directors, thereby increasing the diversity of the board. At the same time, the board of directors must fully consider the protection of major stakeholders when making decisions, especially those with high risks such as technological innovation. As the company’s decision-making level, even if the board of directors did not participate in the implementation of specific company strategic plans, it should strengthen the guidance of the company’s operating direction when making relevant decisions. Boards should also consider the influence of stakeholders on the formulation and implementation of decisions in order to gain their support when the leverage level was high or when creditors were strong in bargaining power (van Zyl and Mans-Kemp, 2021). Based on the above, we have extracted the relevant theoretical framework of board governance and analyzed the role of board governance as shown in Figure 1.

### Resource dependence theory

Grabowska and Otola (2019) pointed out that the basic idea of resource dependence theory is to regard an enterprise as a collection of resources, and the company’s operating conditions can be explained by the scarcity, uniqueness, differentiation and flow costs of available resources. The premises of the resource dependence theory were: Enterprises have both tangible and intangible resources, which are important production factors and the composition of unique differentiated capabilities; Resources are difficult to replicate between enterprises and the cost of flow is very high; The total amount of resources required by an enterprise is relatively stable. When the supply of internal resources is sufficient, the dependence of enterprises on the external environment will be reduced. These unique resources and capabilities can help enterprises to form core competitiveness, so as to ensure that enterprises can have a lasting competitive advantage (Hessles and Terjesen, 2010).

The main support of resource dependence theory for research on board diversification is that it believes that the resources owned by enterprises are different and heterogeneous, and this level of heterogeneity determines the competitiveness of enterprises. As an internal resource of an enterprise, board capital can reduce the organization’s reliance on external resources on the one hand, and on the other hand, it can provide more heterogeneous resources for the organization and help improve firm performance (Simmons, 2012). Combining the research themes, this paper sort out and selected the relevant contents of the resource dependence theory and the effect of board diversity on firm performance as follows: (1) The higher the level of heterogeneity and scarcity of resources owned by a company, the stronger its competitive advantages will be. The company’s operation is composed of behaviors that determine the allocation of resources, and the reserve resources owned by the company will affect the next strategic decision. When a company has high-level, scarce and differentiated resources, it will improve the effectiveness of decision-making, help decision-makers make rational next-step strategic decisions, and form unique competitive advantages (Nam et al., 2018). (2) The resource is impossible or extremely difficult to imitate. The fundamental of a company’s competitive advantage lies in having special resources that are difficult to replicate, so it can bring a long-term competitive advantage to the company. Compared with external resources, the transfer of internal resources is more difficult due to opportunity cost, path dependence and other reasons. Therefore, internal resources are the core competitive capital (Panicker and Upadhyayula, 2020).

### Technical innovation theory

The technical innovation theory pioneered by Schumpeter and his followers, based on “innovation,” revealed the general characteristics of modern economies and the social drivers of their development (Butler, 1988). Schumpeter’s favorite was the entrepreneur. In his view, the entrepreneur was the personification of technological innovation activities. They seek advantages and avoid disadvantages, weeding out the old and distributing the new, and promoting the tide of technological innovation. The existence of entrepreneurial groups was a prerequisite for promoting innovative development and social progress (Cantner et al., 2017). Li et al. (2021) believed that technological innovation can improve the competitive advantage of enterprises and improve the level of company operation. The main body of innovation in technological innovation activities is entrepreneurs or organizations and institutions that play the role of entrepreneurs. There are two fundamental sources of motivation for innovation, one is the pursuit of profit, and the other is the unique entrepreneurial spirit (Turker and Ozmen, 2021). The research of O’Sullivan (2000) pointed out that if an enterprise wants to implement a technological innovation strategy, it needs to meet three conditions: long-term financial commitment, insider strategic control, and effective integration of organizational resources. The implication of financial commitment was that the innovation of the enterprise requires continuous investment of resources. Since innovation involves the original development, reintegration and optimal utilization of production materials, and its asset investment return cycle is relatively long, it needs to obtain a long-term continuous capital investment for innovation decision-making, that is, a financial commitment to innovation. The strategic control of insiders emphasized that insiders have the right to allocate and distribute various resources within the company. Therefore, it can be ensured that the investment behavior of the owner of the company’s resources is not short-sighted, but they will invest in innovation because of the expectation of long-term benefits instead (Parker, 2008). At the same time, innovation needs to consume a lot of resources of enterprises. Only insiders can reallocate these vital resources (Lin, 2016). The effective integration of organizational resources requires companies to effectively allocate complex internal production relations and appropriately motivate innovative personnel to ensure the continuity of innovation (Pan et al., 2018). It can be seen that corporate innovation is a complex process that requires stringent conditions. Nguyen and Dang (2020) believed that innovation imposed higher requirements on organizational subjects than other organizational behaviors, that is, these subjects must not only take risks, but also invest in irrevocable assets. According to the stakeholder theory, the stakeholder who meets the above conditions is the group of board members of the company. Based on the above correlation analysis and logical reasoning between the board of directors and innovation decision-making, it can be seen that the board of directors is a typical representative of the strategic decision-maker, capital investor, risk taker and beneficiary of excess profits in technological innovation. This is also consistent with the findings of Robeson and O’Connor (2013). Based on the above, we believed that the board of directors, as the representative of shareholders’ interests, its members are the main body of enterprise technology innovation under the framework of corporate governance. The above analysis also highlights the theoretical basis for the role of board diversity in the company’s technological innovation behavior.

**Figure 2:**
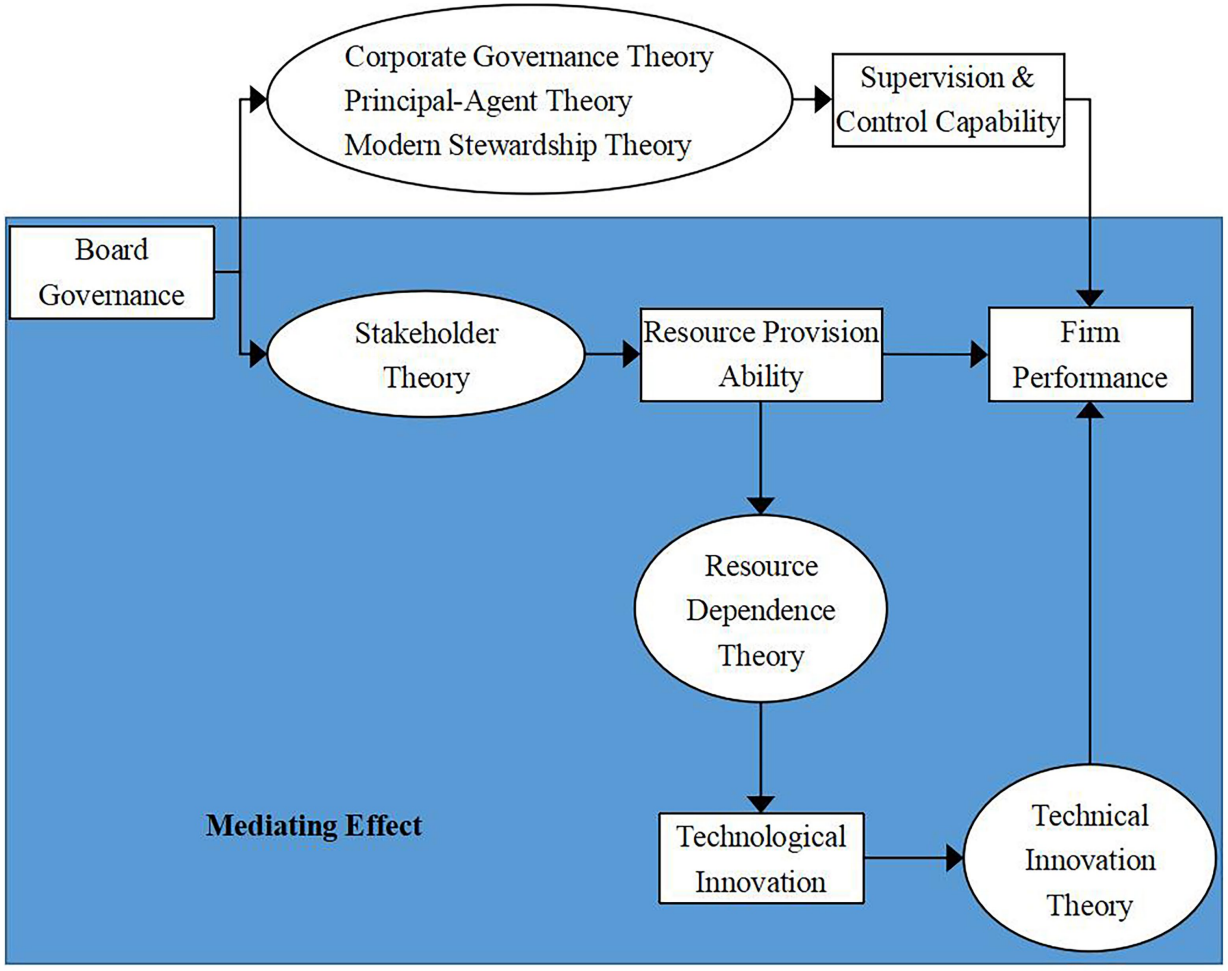
Theoretical framework.

Based on the above theoretical analysis, we draw the overall theoretical logical framework of this paper as shown in Figure 2.

### Research hypotheses

**Figure 3:**
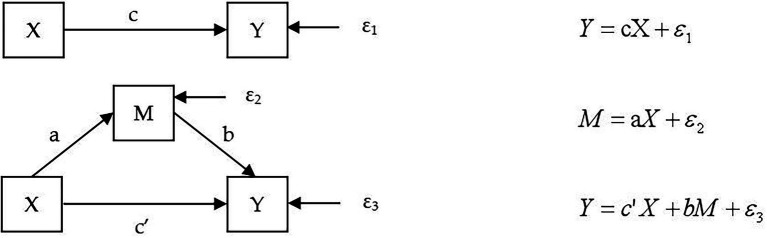
Mediating effect model.

Although the existing measurement software has been able to directly deal with the mediating effect, it is difficult to intuitively reflect the identification process of each stage of the mediating effect. Referring to the mediating effect test method in the field of psychology, this paper adopted the most widely recognized causal step method, that is, the mediating effect was tested in three steps, as shown in Figure 3. This method has been recognized and widely used by a large number of psychologists (Coman et al., 2017; Fairchild et al., 2019; Liu et al., 2021). Compared with measurement software, it can better reflect the intermediate process and internal mechanism of mediating effect, highlighting causal links (Intasao and Hao, 2018; Zhao et al., 2020). The explanatory variable in this paper is board diversity (X), the mediating variable is technological innovation (M), and the explanatory variable is firm performance (Y).

#### Board diversity and technological innovation

Referring to the method of Haynes and Hillman (2010), this paper decomposed board diversity into the heterogeneity of board members’ functional backgrounds, occupational backgrounds and part-time jobs. (1) Heterogeneity of board members’ functional backgrounds: Mizruchi (1983) believed that board members can provide enterprises with other resources other than capital, such as expertise, experience and strategic advice. Through these differentiated resources of the functional backgrounds of board members, companies can gain useful advice on organizational innovation. (2) Heterogeneity of board members’ occupational backgrounds: Marvel and Lumpkin (2007) used the heterogeneity of board members’ knowledge and expertise and their educational attainment as variables to study the impact of board human capital on technological innovation. They also believed that the heterogeneity of board occupational backgrounds can promote the technological innovation of enterprises. (3) Heterogeneity of board members’ part-time jobs: Dakhli and De Clercq (2004) proposed that the intra-industry network of board members will have an impact on the technological innovation behavior of enterprises. When companies have more part-time directors, they are more inclined to adopt competitive behavior. O’Hagan and Green (2002) found that the mutual appointment of chain directors among enterprises in the same industry was conducive to the transfer of knowledge and information, and promoted technological innovation of enterprises. Accordingly, we propose the following hypothesis.

*H*_1_: Board diversity is significantly positively correlated with technological innovation.

Since there are three indicators to measure board diversity in this paper, there are three specific assumptions under H_1_.

*H*_1a_: Functional background diversity of board members is significantly positively correlated with technological innovation.

*H*_1b_: Occupational background diversity of board members is significantly positively correlated with technological innovation.

*H*_1c_: Part-time job diversity of board members is significantly positively correlated with technological innovation.

#### Board diversity and firm performance

Based on the theory of organizational heterogeneity, when the membership of a certain organization or department of a company was more heterogeneous, it was more dynamic and advantageous for the entire company (Klein et al., 1995). (Bianchi et al., 2021) studied the heterogeneity of individual CEOs and top management teams. They confirmed that as the heterogeneity of the CEO’s personal capital increases, the ability to adapt to the industry environment is also stronger, and the more confident he is in the formulation and execution of decision-making. Firms with higher levels of heterogeneity in top management teams also have stronger market competitiveness. Fischer and Pollock (2004) found that the greater the differences among board members, the more effective their judgments and decisions tend to be. Heterogeneous leadership teams can help companies improve strategic decision-making in a dynamically changing environment. Therefore, complex corporate operational behavior needs to be planned by a leadership team with cognitive heterogeneity (Nentwig-Gesemann and Cloos, 2021), thereby enhancing the organization’s creativity and competitive advantages, and improving corporate performance. Diversification of the board can provide companies with differentiated decision-making suggestions, expand strategic vision and choice, improve the board’s ability to perform resource provisioning functions, build internal differentiation advantages within the enterprise, and effectively exert the “catfish effect,” thereby promoting the improvement of firm performance. George and Zhou (2001) believed that the heterogeneity of the board of directors enables the board members to have more and more scientific strategic choices when making decisions to deal with complex changes in the market environment, and to better provide resource advantages and strategic support for corporate organizations. Through the above analysis, we propose hypothesis H_2_.

*H*_2_: Board diversity is significantly positively correlated with firm performance.

*H*_2_ also has three specific hypotheses like H_1_.

*H*_2a_: Functional background diversity of board members is significantly positively correlated with firm performance.

*H*_2b_: Occupational background diversity of board members is significantly positively correlated with firm performance.

*H*_2c_: Part-time job diversity of board members is significantly positively correlated with firm performance.

#### Resource provision from board, technological innovation and firm performance

The research of Pfeffer and Salaneik (1979) found that the board of directors can provide the organization with 3 kinds of advantageous resources: consultation and advice, legitimacy, and network (external resources). These resources support companies to innovate and improve their performance.

First of all, the higher the degree of heterogeneity of board members, the stronger their ability to perform advising and consulting functions, and the more appropriate strategic decisions they make, which in turn positively promotes the level of firm performance (Peres, 2019). With the help of their high level of social capital and human capital, directors can bring decision-making and information resource advantages to the enterprise, thereby helping the enterprise to make correct innovation strategic decisions (Almarayeh, 2021).

Secondly, Board diversity helps to enhance the legitimacy of the company and improve the company’s goodwill (Daily and Schwenk, 1996). Diversity of board members represented an inclusive leadership team and sent a signal to the outside world and the market that the company they invest in and lead is legitimate, long-term stable, risk-resistant, reputable and valuable, and worthy of innovation venture capital (Madera, 2018). This was also confirmed by Hermanson et al.’s (2020) research on the relationship between board heterogeneity and company stock price in US listed companies. As mentioned earlier, technological innovation is the most common risky decision a company makes. A diverse board can improve a company’s reputation and boost market confidence in a company’s innovative behavior. This enabled innovation to be accepted by the market and society, thus realizing the steady state of innovation pointed out by Schumpeter’s innovation theory.

Thirdly, board diversity can provide companies with more external communication networks (Chang and Wu, 2021). The transaction cost theory believed that the asymmetry of information and the complexity of transaction objects and procedures caused enterprises to bear more transaction costs. Joh and Jung (2012) believed that a highly heterogeneous board of directors can obtain valuable information in a timely manner, thereby accurately identifying transaction objects and simplifying the transaction process. This can reduce the uncertainty faced by the organization’s operations, thereby effectively reducing transaction costs (including those of the innovation process). This can reduce the uncertainty faced by the organization’s operations, thereby effectively reducing transaction costs (including those of the innovation process). Hillamn et al. (1999) pointed out by examining the US capital market that a diversified board of directors can more easily establish a relationship with the government, and then strive for more supportive policies and enhance shareholder value. Some scholars have found that chain directors play an active role in promoting innovation between firms (Siebert et al., 2007), reducing vertical collaboration and oversight costs (Chung and Lee, 2020), and transferring information (Omer and Al-Qadasi, 2020).

**Figure 4:**
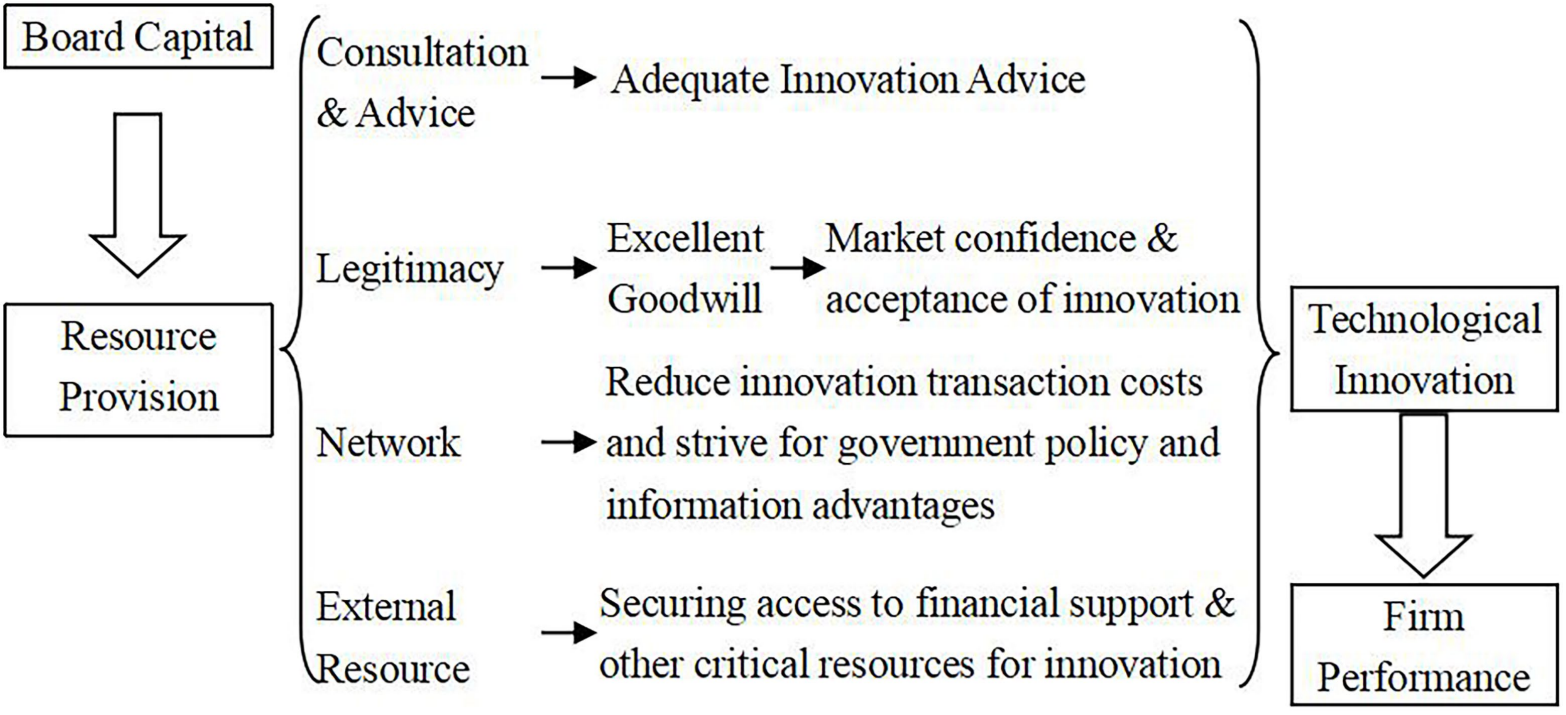
Theoretical analysis diagram of the main research variables.

Based on the above viewpoints, this paper constructed a logical framework for the impact of board diversification on firm performance under the mediating effect of technological innovation, in order to study the internal mechanism of the research variables. The specific theoretical analysis is shown in Figure 4.

Based on the above analysis, this paper put forward the following hypothesis about the relationship between board capital, technological innovation and firm performance.

*H*_3_: Board diversity is significantly positively correlated with firm performance, and technological innovation plays a significant mediating effect.

## Methodology

### Variables

#### Dependent variable: Economic value added

Economic Value Added (EVA) is one of the commonly used measures of firm performance. It is a modified earnings indicator. It is defined as the difference between a company’s capital gain and its corresponding cost, that is, the difference between the company’s net operating profit after-tax and the weighted average cost of capital invested. Milunovich and Tsuei (1996) conducted empirical study of the effectiveness of EVA. He selected indicators such as EVA, ROE, accounting profit and free cash flow for comparative analysis. Through his research, he found that EVA was more closely related to the company’s true value than other indicators. Costa et al. (2019) empirically studied the information increment content of EVA with a sample of listed companies, and confirmed that EVA has greater information content than Operating Cash Flow (OCF) and Residual Income (RI). Since this paper studied the impact of board members, who as owners of residual value, on the company’s performance, EVA was a more suitable evaluation indicator for firm performance. The formula for calculating EVA is:


EVA=NOPAT−(WACC×TC)

The specific elements needed to calculate EVA are shown in Table 1. Even the specific calculation method is given in the paper, in order to remove the noise effect of company stock size, this paper used EVA per-share data, which was directly available from the CSMAR database.

**Table 1:** EVA elements.

Element Name	Abbreviation	Measurement	Resource
Net operating profit after tax	NOPAT	NOPAT = (Net Income - after-tax Non-operating Gains + after-tax Non-operating Losses + after-tax Interest Expense)	Holand and Mathews (2017)
Weighted average capital cost	WACC	WACC = (Equity/(Equity + Debt)) × Cost of Equity + (Debt/(Equity + Debt)) × Cost of Debt × (1-Corporate Tax Rate)	Miles and Ezzell (1980)
Total capital	TC	TC = Total Asset - Current Liability	International Accounting Standards

#### Independent variable: Board diversity

According to the setting of Haynes and Hillman (2010), this paper measured the diversity level of the board from three dimensions: functional background diversity (BDf), occupational background diversity (BDc) and part-time employment diversity (BDp). Among them, we divided the functions of the board of directors into three categories: business resource providers, professional resource providers and public affairs resource providers. Providers of business resources are mainly senior managers within the company, who are good at handling business operations and business negotiations; professional resource providers mostly come from outside the company and provide support in specialized fields such as law, finance, and technology; public affairs resource providers are mainly directors from the government or public institutions, which can provide certain political resources for the company and support for the establishment of good government channels, as shown in Table 2. Our classification of directors’ occupational background draws on the classification criteria of Haynes and Hillman (2010) with appropriate modifications. The occupation of directors is divided into nine categories, namely management personnel, financial personnel, legal personnel, technical personnel, government or community personnel, educational personnel, retirees, freelance personnel, and other personnel (see Table 3). The industry classification of part-time directors referred to the industry classification guidelines of the China Securities Regulatory Commission in 2018. The manufacturing industry is classified according to the second-level code, and other industries are classified according to the first-level code, with a total of 17 industry dummy variables.

**Table 2:** Classification of board members’ functional background.

Board members’ function	Provision of resources from board members
Business resource providers	Provide professional consulting services for the company’s management decisions
	Provide a diverse perspective on internal or external issues of the company
	Representatives from other affiliates
Professional resource providers	(1) Provide legal, financial, professional and public relations advice to the company
	(2) Provide professional services for company mergers and acquisitions
Public affairs resource providers	(1) Provide communication channels for the company, the government, suppliers, etc.
	(2) Provide a non-commercial perspective for corporate governance decisions
	(3) Provide consultation and advice for the company’s public image and public affairs
	(4) Representation of interests of minority shareholders
	(5) The need for capital market supervision

**Table 3:** Classification of board members’ occupational background.

No.	Occupation	Job description
1	Management personnel	Managers at all levels within the company or in affiliated companies
2	Financial personnel	The chief accountant, financial officer, or deputy general manager in charge of accounting in the company, and personnel from accounting firms
3	Legal personnel	Employees working on legal matters in the enterprise, as well as people from law firms
4	Technical personnel	Chief Engineer, Engineer or Technical Director in the enterprise, and persons from professional technical associations
5	Government/community personnel	People working in government departments or industry associations
6	Educational personnel	People from universities or research institutes
7	Retirees	People over the age of 60 who are not working full-time in any unit
8	Freelance personnel	People who work for themselves
9	Other personnel	None of the above

For the calculation of heterogeneity, we use the Herfindahl index to measure the relevant heterogeneity level.


$$H=1-\overset{r}{\underset{i}{\sum }}P_{i}{}^{2}$$

The heterogeneity degree ( H ) of board members’ functions, occupations, and part-time jobs can be obtained by calculation. The higher H is, the higher the diversity of the board is, and vice versa; r represents the number of functions, occupations and part-time types of board members; $P_{i}$ is the ratio of the number of directors of the i-th type to the total number of directors on the board.

#### Mediator: Technological innovation

Academia generally adopted the following methods to measure the innovation ability of enterprises. One was measured by innovation input. Arya and Glover (2006) used R&D expenditure *per capita* to measure the technological innovation capability of enterprises; Liu et al. (2017) use the ratio of R&D expenditure to sales revenue instead measure. Another method is to use innovation output to measure. Sakaki and Jory (2019) used the number of patents or the number of innovative products to measure a company’s innovation capability. In addition, some scholars use the novelty of innovation output to measure innovation capability (Kochhar and David, 1996). However, in view of the poor comparability of innovation results and the influence of external factors such as technology, customers and markets, and less control by management (David et al., 2001), it is not appropriate to use innovation output as an explained variable. Compared with other measurement indicators, R&D investment was mainly determined by the management of the enterprise, which can well reflect whether the operator has agency behavior. At the same time, considering the availability of data, this paper used the ratio of R&D to sales revenue as a proxy variable for corporate technological innovation, denoting it as RD.

#### Control variables

Board capital was also affected by other non-observed variables in the process of influencing firm performance. Therefore, this paper chose to add the following control variables on the basis of previous scholars’ research.

##### Board size

Cheng (2008) found that the expansion of board size will positively affect the business activities of enterprises. On the one hand, the expansion of the board size will bring more knowledge and better complementarity within the board. This is beneficial for enterprises to widely absorb different opinions in the business process and improve the accuracy of business decision-making. The empirical research conclusions of Khalid et al. (2018) showed that board size had a significant correlation with firm value. Haynes and Hillman (2010) also considered board size as one of the control variables in related literature. This paper used the total number of board members as a measure of board size.

##### Equity concentration

Jensen and Meckling (1976) believed that, based on the principal-agent theory, the ownership structure can be used as an important mechanism to guard against the agency behavior of managers. The research of Gul et al. (2010) believed that large shareholders are more inclined to infringe the interests of small shareholders through the tunnel excavation effect. Companies with higher ownership concentration tend to have lower levels of corporate governance (Farooq and Zarouali, 2016). This paper also used it as a control variable, and selects the Herfindahl index (HHI10) of the top ten shareholders for subsequent empirical research.


$$HHI\left(10\right)=\overset{10}{\underset{i=1}{\sum }}\theta^{2}$$

##### Company size

In the field of corporate governance and board governance research, company size was a factor that cannot be ignored. Some scholars used company size as a control variable (Ganda, 2021; Zunac et al., 2021). Considering the influence of magnitude, this paper takes the natural logarithm of the company’s total assets as a control variable.

##### Debt to asset ratio

D’Mello et al. (2018) believed that a company’s debt to asset ratio was a specific manifestation of its solvency and financial policy, and was closely related to whether the company’s operating decisions were aggressive or conservative. The decision of the board of directors was influenced by the debt to asset ratio. If the company’s DA was low, the financial risk it faced was low, and the pressure to repay debts was low. At this time, the board of directors was not subject to external constraints, and the decision-making space was greater. On the contrary, when the company’s DA was high, the company’s creditors facing higher financial risks will intervene more in the company’s decision-making, with a relatively strong position, and the role of the board of directors will be restricted. Therefore, this paper selected the company’s debt to asset ratio as a control variable to join the research model.

##### Company age

Haynes and Hillman (2010) argued that the duration of a company’s existence had a certain degree of influence on board capital. The older the company is, the more mature the corporate governance system and mechanism will be, and the greater the room for the board’s capital to play its role there will be. Therefore, this paper used company age as a control variable. The specific measurement method is the total number of years from the date of establishment of the company to the median year (2019) of the observation interval. The definitions of variables were summarized in Table 4.

**Table 4:** Variables table.

Variable type	Variable name	Abbreviation	Method and description
Dependent Variable	Economic Value Added	EVA	From CSMAR database
Independent Variables	Functional Background Diversity	BDf	See Tables 2, 3
	Occupational Background Diversity	BDc	
	Part-time Job Diversity	BDp	
Mediator	Technological Innovation	RD	R&D/Sales Revenue
Control Variables	Board Size	BS	Total number of board members
	Equity Concentration	EC	Herfindahl Index of Top 10 Shareholders
	Company Size	CS	ln (Total Asset)
	Debt to Asset Ratio	DA	Total Liability/Total Asset
	Company Age	CA	2019-(Year of Company Establishment)

### Data

This paper chose Chinese capital market listed companies as data samples to conduct empirical research. According to the provisions of the “China Company Law,” the term of office of board members shall not exceed 3 years, but the term of office may be re-elected. Accordingly, in order to maintain the continuity of sample observations, this study set the sample time span as 3 years, and the relevant data range from 2018 to 2020, which met the minimum observation interval. In order to avoid the influence of special and extreme company samples on the research conclusions, this paper performed the following screening on the initial research data. (1) We excluded companies with incomplete data disclosure about the variables from 2018 to 2020; (2) The sample listed companies were required to operate continuously from 2018 to 2020; and (3) To ensure the representativeness of the research sample, the research did not include listed companies that were specially treated by the exchange. After screening, 346 valid research samples were finally obtained, with a total of 1,038 panel observations in three years. The data used in this paper were all from the CSMAR database, in which the diversity of the board of directors was collected and sorted manually from the database, listed company reports, and notes. The data processing software was Eviews11.0. The currency measurement unit was RMB. The confidence interval was set to 95%.

### Model

**Figure 5:**
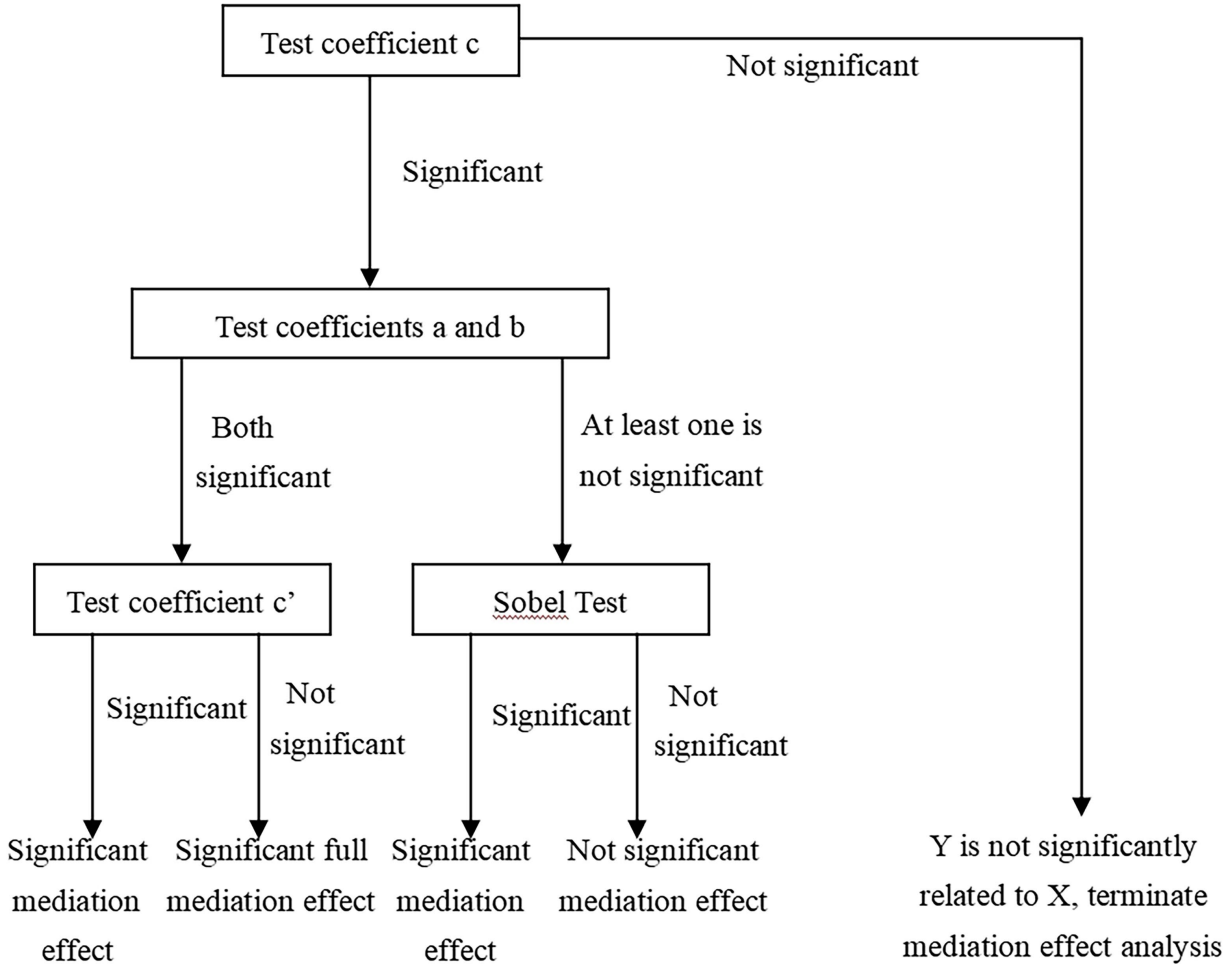
Mediation effect test process.

Figure 5 describes the three steps of mediating test in the field of psychology. Meanwhile, combined with Figure 3, we proposed the modeling path as follows. Firstly, we need to explore the influence of independent variables on the dependent variable. If the coefficient c of each variable is not statistically significant, the mediating effect analysis is terminated directly, and the variable does not play a mediating role. If c is significant, we proceed to the next step of analysis. Secondly, we analyzed the correlation between independent variables and the mediating variable. If the coefficient a is significant, we go to the next step of analysis. If a is not significant, the Sobel test will be performed after the coefficient b is determined in the third step. Thirdly, we analyzed the multivariate correlation among independent variable, the mediator and the dependent variable, and determined the significance of coefficients b and c’. Combined with the analysis results of the second step, if both a and b are significant, the significance of the coefficient c’ is verified. If c’ is significant, it is confirmed that the mediating variable has played a significant partial mediating effect. If c’ is not significant, it is a significant full mediating effect.

According to hypothesis $H_{2}$ and each sub-hypothesis, the empirical model was constructed as follows:


$$\begin{array}{c} EVA_{i,t}=C+c_{1}BDf+c_{2}BDc+c_{3}BDp\\ +c_{4}\sum ControlVariables+\varepsilon_{i,t} \end{array}$$

According to hypothesis $H_{1}$ and each sub-hypothesis, the empirical model was constructed as follows:


$$\begin{array}{c} RD_{i,t}=C+a_{1}BDf+a_{2}BDc+a_{3}BDp\\ +a_{4}\sum ControlVariables+\varepsilon_{i,t} \end{array}$$

According to hypothesis $H_{3}$ , the empirical model was constructed as follows:


$$\begin{array}{c} EVA_{i,t}=C+bRD+c{'}_{1}BDf+c{'}_{2}BDc+c{'}_{3}BDp\\ +c{'}_{4}\sum ControlVariables+\varepsilon_{i,t} \end{array}$$

## Analysis and discussion of results

### Descriptive analysis

Through descriptive statistics, we firstly described the range and degree of dispersion of the sample data, as shown in Table 5. From 2018 to 2020, the average EVA of the sample companies was 0.097 Yuan per share. After considering the exchange rate, there was a gap of 150 times between it and the average value of US listed companies (US$2.11 per share in) in 2002 (Zhang, 2009). The level of residual value creation of Chinese listed companies was not high. In addition, the median EVA was 0.051, indicating that most companies were still creating economic value. However, this value spanned from−4.404 to 2.633, with a standard deviation of 0.437, which indicated the obvious differences in performance between companies. In the perspective of independent variable Board Diversity (BD), the average values were all above 0.299, which showed that the sample companies have deployed board members with different functions, occupations and part-time positions in their management and operation practices. As far as the intermediary variable, technological innovation, the average R&D to sales revenue ratio of the sample enterprises was only 0.026, and the median was 0.001, indicating that the innovation intensity was still insufficient. The standard deviation of RD was 0.246, which showed that the R&D investment of the selected sample companies was quite different.

**Table 5:** Descriptive analysis.

Variables	Average	SD	Median	Min.	Max.
EVA	0.097	0.437	0.051	-4.404	2.633
BDf	0.591	0.054	0.593	0.491	0.681
BDc	0.457	0.080	0.317	0.382	0.623
BDp	0.299	0.212	0.304	0.000	0.675
RD	0.026	0.246	0.001	0.000	0.136
BS	8.760	1.782	9.000	5.000	18.000
EC	0.153	0.114	0.119	0.003	0.664
CS	22.000	1.240	21.788	19.541	26.751
AL	0.403	0.216	0.401	0.011	0.993
CA	14.523	5.273	14.000	3.000	32.000

### Multicollinearity test

In order to ensure the rigor and reliability of the model, this paper conducted collinearity diagnosis on the main research variables to prevent the problem of insufficient goodness of fit and explanatory power of the equation. It can be seen from Table 6 that the tolerance of each variable is above 0.874. And the variance inflation factor (VIF) is below 1.242. This shows that there is no significant multicollinearity problem among the variables, and the explanatory power of the independent variable and the mediator variable to the dependent variable is not affected by noise.

**Table 6:** Multicollinearity test.

Variables	Tolerance	VIF
EVA	0.917	1.131
BDf	0.963	1.104
BDc	0.874	1.242
BDp	0.904	1.231
RD	0.892	1.238

### Regression analysis of mediating effect

In this paper, the random effects panel model, mixed regression model and fixed effect panel model were used to perform regression analysis on the relevant variables for the observed samples through Eviews11.0 software. After testing the different models by Hausman test, we decided to use the cross-section (C-S) weighted fixed-effects panel model to empirically process the data. According to the three steps of the psychological mediating effect test and the three hypotheses and models proposed above, the observed variables were introduced one by one. Among them, the dependent variable of model 2 was RD, and the dependent variable of the other models was EVA. The details are shown in Table 7.

**Table 7:** Results of the three-step mediating effect test.

	Model 1(EVA)	Model 2 (RD)	Model 3 (EVA)
BDf	0.457^**^	0.037^***^	0.040^*^
BDc	0.116^**^	0.023^**^	0.107
BDp	0.085^***^	0.031^**^	0.042^**^
RD	–	–	0.113^**^
BS	−0.006	−0.003	−0.001
EC	−0.316^***^	−0.313^***^	−0.311^***^
CS	0.098^***^	0.075^***^	0.096^***^
AL	−0.643^***^	−0.649^***^	−0.647^***^
CA	2.59E-05	10.2E-05	5.33E-05
Adjusted R^2^	0.418	0.403	0.508

^*^, ^**^, ^***^ represent significance at the 10, 5, 1% level, respectively.

#### Model 1 analysis: Board diversity and firm performance

Firstly, the regression results of Model 1 show the effect of BDf on EVA. It can be seen that there is a significant positive correlation between the two at the 95% level, which is consistent with the findings of Hillman and Dalziel (2003). This shows that the heterogeneity of the functional background of board members is often manifested in the heterogeneity of directors’ experience and resources. This is conducive to generating first-mover advantages such as strategic change, competitive behavior, etc., and improving the company’s performance level. At the same time, a board with a rich functional background can more accurately evaluate and supervise the CEO and his management team, accurately evaluate the effectiveness of strategy execution, and identify important resources and opportunities (Francis et al., 2019). The hypothesis H_2a_ proposed in this paper has been empirically verified.

Secondly, the results of Model 1 also reflect the effect of BDc on EVA. It can be seen that there is a significant positive correlation between the two at the 95% confidence level, which is consistent with the findings of Haynes and Hillman (2010) and Arayakarnkul et al. (2022). This shows that the greater the occupational differences among board members, the higher the company’s performance. The board members come from different industries and can provide different opinions on the decision-making of the board of directors from multiple angles and multiple levels, which ensures the optimization of strategic decision-making. Hypothesis H_2b_ has been verified.

Finally, the regression results show the effect of BDp on EVA. It can be seen that there is a significant positive correlation between these two variables at a confidence interval of 99%. This is consistent with the findings of Ahrens and Scheele (2022). Board members work as part-time directors in different industries, which makes it easier for them to have access to a broad range of knowledge and management experience and broaden their horizons. This is conducive to resource interaction with business partners outside their industry such as upstream and downstream companies in the production chain, improving transaction efficiency, reducing transaction costs, and thus improving firm performance. Hypothesis H_2c_ is empirically verified.

In empirical research, sometimes variables may be significantly correlated but do not necessarily have an economic causal relationship (Sneed, 1968; Cepni et al., 2022). In order to test the economic value of the hypotheses about the impact of board diversity on corporate performance, and to test whether there is a mediating effect, this paper used the Granger causality test. The dependent variable is the firm performance proxy variable EVA. The independent variables are the sub-index variables of board diversity. The results of the causal analysis are shown in Table 8.

**Table 8:** Granger test results.

**Independent variables**	**Chi** ^**2**^	***p*-value**
BDf	0.159	0.853
BDc	0.890	0.306
BDp	3.507	0.043^**^

** Represents significance at the 5% level.

It can be seen from Table 8 that among the sub-indicators, only the part-time heterogeneity of board members (BDp) and firm performance (EVA) showed a Granger causal relationship, and there was only a mathematical statistical relationship between the remaining variables and firm performance. This shows that although some indicators of board diversity (BDf and BDc) were significantly related to corporate performance, this effect was not direct in economic meaning and requires a mediating variable. It is worth noting that, according to the test logic of the mediation effect proposed above, the above three independent variables representing board diversity have a significant impact on the dependent variable. Therefore, a further test of the mediating effect can be carried out.

#### Model 2 analysis: Board diversity and technological innovation

Firstly, the results of Model 2 illustrate the impact of the functional background heterogeneity of board members on technological innovation. It can be seen that there is a significant positive correlation between BDf and RD at the 99% confidence interval, which is consistent with the findings of Sternberg (2000) and Makkonen (2022). The higher the degree of functional heterogeneity of board members is, the more professional and diversified board governance in the enterprise will be, which can provide enterprises with better professional consulting functions, thereby improving the level of enterprise innovation decision-making. Hypothesis H_1a_ has been verified.

Secondly, BDc and RD are significantly positively correlated at the 95% confidence interval, that is, the diversity of the occupational background diversity of board members can positively promote the company’s technological innovation. Hypothesis H_1b_ has been verified. The research of Zanoni and Janssens (2015) and Wang (2022) found that innovation includes the generation of new ideas, the identification of new opportunities, and the formulation and implementation of new decisions, all of which place high demands on the identification, cognition and discovery of opportunities. Due to the addition of different knowledge and viewpoints, the knowledge and expertise of decision-makers tend to be heterogeneous, which help them better identify innovation opportunities and broaden their innovation horizons. This facilitates the generation and execution of high-quality innovation decisions. On the contrary, if the occupational background of the board becomes homogeneous, then the board tends to be conservative and is not good at identifying and discovering new opportunities. This is not conducive to the company’s technological innovation.

Thirdly, BDp and RD are significantly positively correlated at the 95% confidence interval, which is consistent with the research conclusions of most scholars (Hillman et al., 2000; Wincent et al., 2010; Nadeem et al., 2020). As part-time directors in different industries, board members will broaden their horizons, better identify and discover the excellent innovation cultivation models and innovation methods of other companies, and seize innovation opportunities, thereby improving the company’s technological innovation level. Hypothesis H_1c_ has been verified. These three groups of significant correlation also mean that the respective variables and mediating variables can enter the third step of verification.

#### Model 3 analysis: Final test of mediating effect

We were surprised to find that after adding the mediator variable RD, the correlation between BDf, BDc and EVA changed from a significant positive correlation (Model 1) to no correlation (Model 3). According to the validation criteria of the mediating effect in Figure 5, this means that RD played a fully significant mediating role in the process of BDf and BDc positively affecting EVA. That is to say, the functional background heterogeneity and occupational background heterogeneity of board members affect EVA through technological innovation. Meanwhile, RD played a partial mediating role in the process that BDp positively affecting firm performance. In addition, the significance level of RD also passed the test. Based on the above, according to the test logic of the mediating effect (Figures 3, 5), it can be concluded that the board diversity has a significant positive correlation with firm performance, and technological innovation plays a significant mediating effect.

#### Control variables and goodness of fit of the model

From the regression results of the three groups of models in Table 7, control variables such as board size (BS) and company age (CA) have no significant impact on firm performance and technological innovation. Equity concentration (EC) and debt to asset ratio (DA) are significantly negatively correlated with EVA and RD. Company size (CS) was significantly positively correlated with the dependent variable and the mediator. The significant verification results of the above control variables are in line with the research results of Abdullah et al. (2019), Sheng and Shen (2020), and Gomes et al. (2009) respectively. From the comparison of R^2^ of model 1 and model 3, after adding the mediating variable, R^2^ of model 3 increases significantly, which indicates that the model with mediating effect has a higher goodness of fit.

### Robustness test

In this paper, the Chow mutation point test is used to verify the robustness of the regression model. Taking 2019 as the mutation point, models 1.1 and 1.3 are randomly selected for robustness testing, and the test analysis Table 9 is obtained.

**Table 9:** Robustness test for BD & EVA, and mediating effect model.

**BD&EVA**			**Mediating effect model**		
**Statistics**	**Value**	***p*-value**	**Statistics**	**Value**	***p*-value**
F	23.122	0.000	F	19.431	0.000
LR	12.141	0.000	LR	10.201	0.000
Wald	40.244	0.000	Wald	37.173	0.000

As shown in Table 9, after adding the 2019 data as the mutation point, the value of *p*s of the F statistic, LR statistic and Wald statistic of the model are all extremely small. So we rejected the null hypothesis. After adding mutation points, the model did not change significantly, and the robustness of the two models passed the test. Through robustness tests, we examined the explanatory power and stability of the mediator variables. The above results also showed that after changing the sampling samples, the original evaluation methods and indicators still maintained a relatively consistent and stable interpretation of the evaluation results.

### Contribution analysis of the mediator

**Figure 6:**
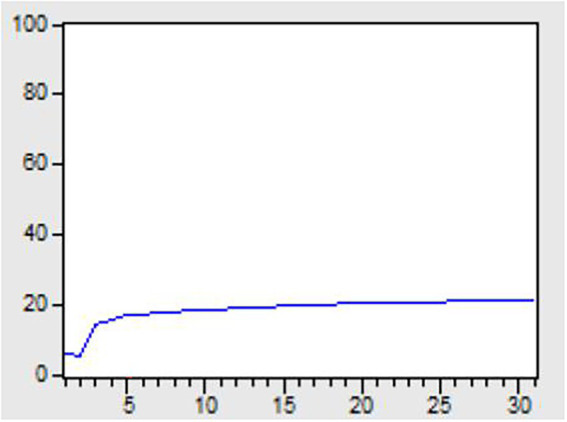
Percent EVA variance due to RD.

In order to empirically analyze the impact and contribution of the mediator variable, technological innovation, to firm performance, this paper adopted impulse response analysis method, and used dynamic variance decomposition method to explore and determine the impact degree, and further explained the impact degree of explanatory variables on the explained variables. The data processing method is to select the average value of RD and EVA of 346 sample companies, and use Eviews software to high-frequency smooth the annual data into monthly data. Finally, 3 times 12, a total of 36 groups of pulse data are obtained, and the variance is decomposed. The results are shown in Figure 6.

As shown in Figure 6, the solid line is the part of the variance of EVA due to RD. The impact of technological innovation on firm performance gradually increased and converged to 20% after a time lag of about 5 months. This shows that 20% of the EVA variance in the model can be explained by RD. The effect is relatively significant, and the mediating variable has a certain degree of contribution. At the same time, the results of this paper on the hysteresis of the impact of technological innovation on firm performance are similar to those of Kniazevych (2010) and Lam et al. (2017).

## Conclusion and discussion

### Research conclusions and findings

Based on theoretical analysis and empirical research, this paper identified technological innovation as a mediating variable in three stages, which has a significant full mediating effect in the process of board diversity affecting corporate performance. Through the serial analysis of “board diversity” — “technological innovation” — “firm performance,” the complex internal relationship among the three was revealed. This illustrated the important mediating role of technological innovation. We empirically studied the mechanism by which board diversity affects corporate performance, and opened the “black box” of the entire chain of action.

Our main conclusions are that, firstly, board diversity can significantly improve a company’s technological innovation level and performance. a. The heterogeneity of the functional background of board members is often manifested in the heterogeneity of experience and resources owned by directors, which will increase the scope of corporate strategic choices and decision-making. This is conducive to generating advantages such as strategic change, competitive behavior, technological innovation, and thus improving the company’s performance level. At the same time, boards with rich functional backgrounds can more accurately assess and monitor CEOs and their management teams. These board members can also accurately evaluate the effectiveness of innovation strategy execution and identify important resources and opportunities. b. The greater the occupational differences among board members, the higher the firm’s performance. Board members come from different industries and can provide different opinions on board decisions from multiple perspectives and levels, thus ensuring the optimization of innovative strategic decisions. c. Board members work as part-time directors in different industries, which makes it easier for them to gain access to broad knowledge and management experience and broaden their horizons. This facilitates resource interaction with external partners such as upstream and downstream companies in the same value chain, thereby improving transaction efficiency, reducing transaction costs, and improving firm performance. This has also passed the Granger causality test, proving its economic connotation. Secondly, the connection between board diversity and firm performance is based on technological innovation, that is, board diversity promotes firm performance by improving the level of technological innovation. Technological innovation played a complete mediating effect on the heterogeneity of functional background and occupational background, and also played a significant partial mediating effect on the heterogeneity of part-time employment.

### Research contributions and limitations

The theoretical and practical contributions that this paper attempted to make mainly include the following aspects. We found that while supporting existing theoretical analyses with empirical research, open up the “black box” of the relationship between board diversity and firm performance. We comprehensively examine the complete internal impact path of board diversity on firm performance of “capital investment” - “decision-making process” - “performance output.” We proved that board diversity is a potential internal cause of firm performance improvement. This internal cause needs to be reflected in decision-making before affecting corporate performance. This paper provided a new understanding of existing theoretical and empirical research models from the perspective of the mediating effect of technological innovation. All of these improve the theoretical system of board diversity governance and make board governance theory more systematic. This study also expanded the research extension of related theories such as technological innovation, and at the same time provides some empirical research references for academics and practitioners.

We suggest that companies can design appropriate incentive systems and introduction policies which may attract directors with diverse functional, occupational and part-time backgrounds to the board. This will increase the level of board diversity and optimize the membership and overall structure of the board. While enhancing the governance vitality of the board, it may also promote them to actively exert their resource advantages in terms of expertise, knowledge and skills. These can promote the company’s technological innovation and firm performance.

There are some limitations of this paper and prospects for future research. This paper used public data of listed companies rather than survey data. But board diversity is a refocus of board governance. The focus is on examining the “human” factor. Data distortion will have a greater impact on the effective measurement of board diversity. Therefore, follow-up research can obtain first-hand data of board members through in-depth company visits and questionnaires, so as to more accurately measure the capital level of the board of directors. At the same time, in further research, case study may be used to provide representative research conclusions.

## Data availability statement

The raw data supporting the conclusions of this article will be made available by the authors, without undue reservation.

## Author contributions

JA and HD were responsible for writing the initial draft of the manuscript and putting forward the main propositions. HD was responsible for further modification and improvement of the manuscript. MY was responsible for reviewing and editing the manuscript. All authors contributed to the article and approved the submitted version.

## Funding

This research was funded by the National Natural Science Foundation of China (No. 72091313).

## Conflict of interest

The authors declare that the research was conducted in the absence of any commercial or financial relationships that could be construed as a potential conflict of interest.

## Publisher’s note

All claims expressed in this article are solely those of the authors and do not necessarily represent those of their affiliated organizations, or those of the publisher, the editors and the reviewers. Any product that may be evaluated in this article, or claim that may be made by its manufacturer, is not guaranteed or endorsed by the publisher.
